# Protein Language Models and Machine Learning Facilitate the Identification of Antimicrobial Peptides

**DOI:** 10.3390/ijms25168851

**Published:** 2024-08-14

**Authors:** David Medina-Ortiz, Seba Contreras, Diego Fernández, Nicole Soto-García, Iván Moya, Gabriel Cabas-Mora, Álvaro Olivera-Nappa

**Affiliations:** 1 Departamento de Ingeniería en Computación, Universidad de Magallanes, Punta Arenas 6210005, Chile; 2 Centre for Biotechnology and Bioengineering, CeBiB, Universidad de Chile, Santiago 8370456, Chile; 3 Max Planck Institute for Dynamics and Self-Organization, Am Faßberg 17, 37077 Göttingen, Germany; 4 Departamento de Ingeniería Química, Universidad de Magallanes, Punta Arenas 6210005, Chile; 5 Departamento de Ingeniería Química, Biotecnología y Materiales, Universidad de Chile, Santiago 8370456, Chile

**Keywords:** antimicrobial peptides, machine learning, protein language models, generative learning, peptide discovery, peptide design

## Abstract

Peptides are bioactive molecules whose functional versatility in living organisms has led to successful applications in diverse fields. In recent years, the amount of data describing peptide sequences and function collected in open repositories has substantially increased, allowing the application of more complex computational models to study the relations between the peptide composition and function. This work introduces AMP-Detector, a sequence-based classification model for the detection of peptides’ functional biological activity, focusing on accelerating the discovery and de novo design of potential antimicrobial peptides (AMPs). AMP-Detector introduces a novel sequence-based pipeline to train binary classification models, integrating protein language models and machine learning algorithms. This pipeline produced 21 models targeting antimicrobial, antiviral, and antibacterial activity, achieving average precision exceeding 83%. Benchmark analyses revealed that our models outperformed existing methods for AMPs and delivered comparable results for other biological activity types. Utilizing the Peptide Atlas, we applied AMP-Detector to discover over 190,000 potential AMPs and demonstrated that it is an integrative approach with generative learning to aid in de novo design, resulting in over 500 novel AMPs. The combination of our methodology, robust models, and a generative design strategy offers a significant advancement in peptide-based drug discovery and represents a pivotal tool for therapeutic applications.

## 1. Introduction

Peptides are versatile, bioactive, short amino acid chains, with lengths of 5–15 amino acids and rarely exceeding 30 amino acids [1,2]. The diverse roles that they play in living organisms, e.g., acting as structural components, enzymatic inhibitors, hormones, host defense molecules, neurotransmitters, and cell surface receptors, have attracted great research interest due to their potential applicability in the biochemical and pharmaceutical industries [3,4,5,6]. Of particular interest, some peptides may possess antimicrobial properties, including antibacterial, antiviral, antifungal, and antiparasitic effects [7].

The increasing number of peptides documented in the literature has provoked significant interest in applying computational biology techniques to analyze peptide sequences, predict biological activity, calculate physicochemical properties, and assist in peptide design [8,9]. Predictive models, including machine learning and deep learning architectures, have been developed for tasks such as antimicrobial and antiviral peptide classification [10,11,12,13,14,15]. Generative models have also been employed to aid therapeutic peptide design by generating novel peptide sequences. Techniques such as variational autoencoders (VAE) and generative adversarial networks (GAN) have shown promise in creating new antimicrobial peptides [12,16,17,18,19,20,21,22]. Recent advances in diffusion models have further enhanced the generative approaches for antimicrobial peptide design [23,24,25].

This work implements AMP-Detector, a sequence-based functional classification model, to assist the discovery of potential antimicrobial peptides. This work implements 21 binary classification models to predict the functional biological activity of peptide sequences, including antimicrobial, antibacterial, and antiviral functions. The proposed pipeline for the training of these predictive models integrates numerical representation strategies, machine learning algorithm exploration, statistical approaches to select optimal combinations, Bayesian methods for hyperparameter tuning, and the criteria-based selection of the best models [26]. On average, the models achieved precision of over 83% and a Matthews correlation coefficient of 0.7 across the explored tasks. New peptides for all types of functional biological activity evaluated in this work were discovered using the trained models and the Peptide Atlas database [27]. Additionally, over 100,000 peptides were generated using variational autoencoder (VAE) approaches, and their functional biological activity was evaluated with the trained binary classification models, leading to the in silico discovery of more than 600,000 potential antimicrobial peptides, 200,000 antibacterial peptides, and 500,000 antiviral peptides. AMP-Detector incorporates an inference tool to facilitate the evaluation and exploration of unknown peptide sequences with potential antimicrobial peptides. Integrating trained classification models with generative approaches demonstrates the efficacy of the implemented methods in annotating and designing novel potential antimicrobial peptides, showing clear advantages over traditional computational biology approaches.

## 2. Results and Discussion

### 2.1. Main Features of the Studied Datasets

**Figure 1:**
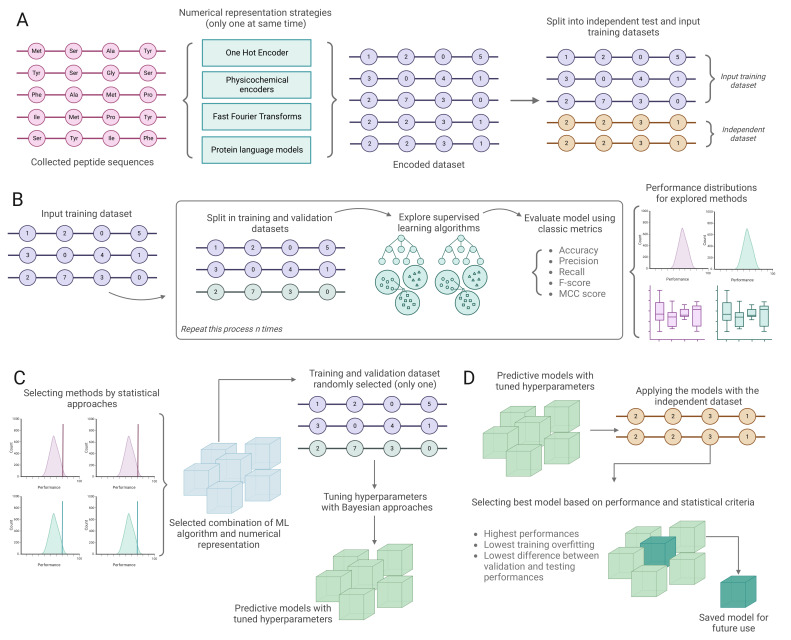
**Proposed methodology to generate and evaluate predictive models.** (**A**) Numerical representation of sequence datasets. Here, we explore different encoding strategies, including classic methods such as one-hot encoders, physicochemical property-based encoders, and embedding based on pre-trained models. All different methods are applied individually. Once the input dataset is encoded, it is randomly split in a 90:10 ratio, using the first part to develop models and the second as a benchmark dataset. (**B**) Using the model development dataset and all of its possible numerical representations, we explore different 80:20 partitions to use for model training and validation. We explore and evaluate different models and hyperparameters using classic performance metrics. As this stage is repeated an arbitrary number of times, we obtain distributions of performance for each model. (**C**) Based on the distribution of performance, the best-performing combinations of algorithms and numerical representations are selected based on statistical criteria. These models undergo a hyperparameter optimization procedure based on Bayesian criteria. (**D**) Finally, we evaluate the performance of the models generated (and other tools/methods used to compare them) using the benchmark dataset and export the best strategy for future use.

We collected more than 100,000 peptide sequences with reported functional biological activity from Peptipedia [28] and applied a filter to remove all peptide sequences containing non-canonical residues, resulting in the selection of 86,477 peptides for the development of predictive models following the pipeline described in Figure 1. Tasks such as antimicrobial, antibacterial, antiviral, antiparasitic, antifungal, and antimammalian cell processes had more than 5000 positive examples. In contrast, after redundancy evaluation, the antimalarial and quorum-sensing tasks had less than 200 positive examples. The low number of examples in these tasks could negatively affect the performance of the trained models, as these datasets are typically categorized as Low-N datasets. Consequently, more sophisticated machine learning strategies, such as transfer learning or contrastive learning methods, are necessary to achieve optimal performance [29].

For each task, besides encoding using the seven pre-trained models detailed in the table below, we included the one-hot encoding approach, eight physicochemical-based encoding methods, and eight Fast Fourier Transform (FFT)–physicochemical representations. Altogether, we considered 24 encoding techniques for each task. Methods like ProTrans ALBERT and one-hot encoding generate vectors with the highest dimensionality. In contrast, methods based on physicochemical properties and FFT aim to represent and characterize a function using a lower dimensionality space (See Section S4 of the Supplementary Materials for more details).

### 2.2. Binary Classification Tasks

The proposed pipeline, summarized in Figure 1, was utilized to train binary classification models for each of the 21 types of biological activity examined in this study. Over 15,000 models were successfully trained by exploring various numerical representation strategies, supervised learning algorithms, and hyperparameter configurations. Each combination was evaluated using classic metrics such as precision and recall. Subsequently, statistical criteria were applied to identify the optimal numerical representation strategy and supervised learning algorithms, followed by a detailed hyperparameter tuning process. The performance was then assessed using an independent test dataset, allowing for the selection of models with the highest performance and the lowest overfitting rate. This section summarizes the statistical analysis conducted during the exploration stage, the chosen combinations for the hyperparameter tuning process, and the tuned models generated for each biological task. Additionally, a benchmark analysis of the most common biological activity types evaluated in this study is included to demonstrate the efficacy of the proposed methodology.

#### 2.2.1. Performance Statistics

Our model training and validation pipeline outputs several performance metrics used for model selection. The training performance reports the mean over several *k*-cross-validations in 80% of the dataset, while the validation performance is a single value obtained when applying the generated model on the 20% remaining. As the 80:20 partition is repeated 30 times and the results are aggregated over different categories, we obtain distributions instead of single performance values.

**Figure 2:**
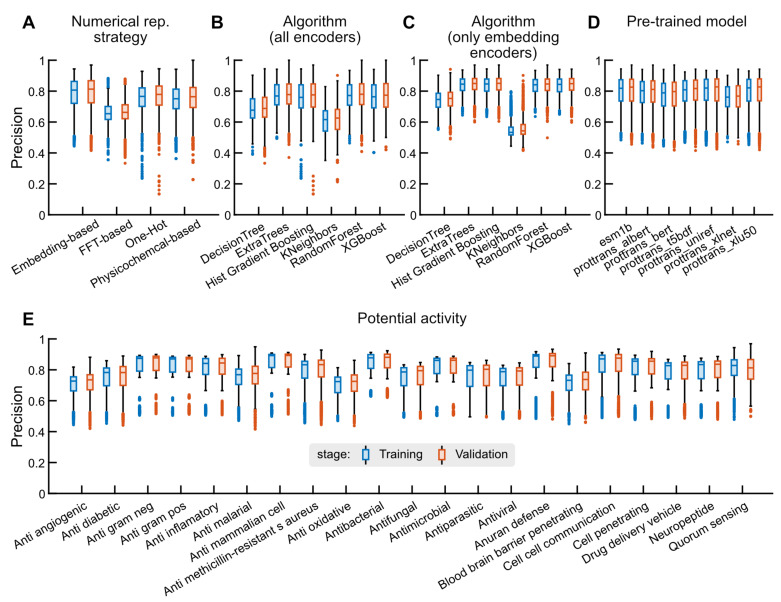
**Performance distributions in the model exploration stage.** Our model training and validation pipeline outputs several performance metrics, which are used for model selection. The training performance reports the mean over several k-cross-validations in 80% of the dataset, while the validation performance is a single value obtained when applying the generated model on the 20% remaining. As the 80:20 partition is repeated several times and the results are aggregated over different categories, we obtain distributions instead of single performance values. In blue, the distribution of the mean performance in training is narrower than the distribution in validation (orange) as a consequence of the central limit theorem. We present these performance measures for different numerical representation strategies (**A**), supervised learning algorithms on the whole dataset (**B**) and supervised learning algorithms filtering for only embedding-based encoders (**C**), for embedding representation through pre-trained models (**D**), and for the different classification tasks (**E**).

Models trained on datasets encoded using embedding representations perform better than models trained on datasets encoded using one-hot encoders, physicochemical-based encoders, and FFT-based encoder strategies. The embedding representation achieves mean precision of 77% during training and testing. In contrast, while physicochemical-based and one-hot-based encoders present similar average precision (75%), they exhibit more outliers and erratic behavior in distribution. FFT-based encoders show the lowest mean precision (67%) but do include positive outliers (see Figure 2A). Regarding algorithms, ensemble-based methods like Random Forest and ExtraTrees achieve the highest mean precision at 77%. In contrast, methods like decision trees and nearest neighbors obtain lower performance, with precision values below 70% (see Figure 2B).

Given that the performance distribution for models using embedding-based representations achieved, on average, the highest performance, we extended the evaluation to include supervised learning algorithms and embedding representations. Generally, models based on ProTrans T5 Uniref, ProTrans T5 xlu50, and Esm1B achieve the highest performance across all evaluated metrics. However, except for ProTrans t5 BERT, there are no significant differences in the average performance of each pre-trained model used in this work (see Figure 2C). Additionally, the performance distribution by algorithm shows that ensemble-based models remain the best-performing supervised learning algorithms compared to decision tree or *K*-nearest neighbors approaches (KNN) (see Figure 2D).

When evaluating the performance by activity, on average, the trained models for anti-Gram(+), antimammalian cell, anuran defence, anti-inflammatory, and cell–cell communication tasks show the highest average precision, with values above 78%. These tasks also exhibit the highest sensitivity (greater than 0.77) and MCC performance (greater than 0.55). In contrast, antiangiogenic, blood–brain barrier penetration, and antioxidative peptide identification exhibit the lowest average precision, with values below 67% (see Figure 2E).

#### 2.2.2. Optimizing the Hyperparameters of Selected Models

Upon completing the exploration stage and selecting the best-performing models, we obtained 87 combinations of supervised learning algorithms and numerical representation strategies, which were chosen for the hyperparameter optimization process. The most commonly selected combinations were ProTrans xlu50 with ExtraTrees (8.7% of selected combinations), ProTrans T5 Uniref with ExtraTrees (8.7%), and Esm1B with ExtraTrees (7.8%). Less frequently selected combinations, such as ProTrans T5BDF with Random Forest or Histogram-Based Gradient Boosting, represented only 0.87% of the selections. Combinations like ProTrans ALBERT with Hist Gradient Boosting or XGBoost and ProTrans BERT with all supervised learning algorithms except ExtraTrees did not achieve the cutoff performance and thus were not observed among the selected sample.

We followed a Bayesian approach to determine the optimal set of hyperparameters for the 87 selected combinations of models, evaluating 50 trials per combination, with a specific random state (random state = 42) applied to all evaluations. The performance estimation followed the same procedure as in the exploration stage. An independent dataset was also used to assess the models’ performance with tuned hyperparameters and to compare all evaluated methods. Tasks like antifungal, antiviral, and anti-inflammatory had more than seven possible combinations explored, while models for cell-penetrating, antimammalian cell, and blood–brain barrier penetration properties considered only one combination (see Section S5 of the Supplementary Materials for more details).

Using the evaluation criteria proposed in this study, the best classification models were selected with the highest performance, the lowest overfitting during training, and the smallest differences between the validation and testing performance. Table 1 summarizes the performance of the selected models for each task, covering the training, validation, and testing stages. Classification models for anuran defense, quorum sensing, antibacterial activity, cell–cell communication, and antimammalian cell activity achieved precision values exceeding 90% across the training, validation, and testing stages. Only the models for antiviral and antimalarial tasks achieved performance below 80% in the testing stage. The remaining activity types maintained precision values over 80%. Hyperparameter tuning improved the testing performance in 13 out of 21 cases. In five cases, the performance remained consistent with the validation and training phases, and, in three cases—antimalarial, antiviral, and antidiabetic—the performance slightly decreased. Despite these reductions, the lowest precision value was 78%, demonstrating the proposed methodology’s robust capabilities to generalize the training of functional classification models.

Analyzing the recall or sensitivity performance of the models, those with the highest precision also reported the highest recall values, achieving sensitivity scores above 0.9. In contrast, the blood–brain-barrier-penetrating, antiviral, and antimalarial classification models had the lowest recall values, with a sensitivity score of 0.78 (see Section S6 of the Supplementary Materials for more details).

The 21 highest-performing models were employed to develop AMP-Detector, a Python library designed to enhance the usability of trained models by facilitating the inference of biological activity for peptide sequences. AMP-Detector integrates both the trained and pre-trained models for embedding representation, streamlining the execution of all steps in the pipeline.

An exploration of four deep learning architectures was conducted to compare the performance of the proposed models with predictive models trained using neural network approaches. The explored architectures included a Convolutional Neural Network (CNN), Bi-Long Short-Term Memory (Bi-LSTM), the Bi-Gated Recurrent Unit (GRU), and a combination of the CNN and LSTM architectures (see Section S8 of the Supplementary Materials for more details). All tasks were evaluated using the selected embedding representation and the same datasets for training, validation, and testing.

We calculated the model performance for each of the architectures considered during both the validation and testing stages. Deep learning architectures generally showed higher performance than the proposed pipeline during validation. However, during testing with the independent dataset, the deep learning models exhibited lower performance in most cases compared to the implemented models using AMP-Detector. This discrepancy between the validation and testing performance suggests an overfitting problem with the deep learning models.

In specific cases, such as antimicrobial, cell–cell communication, cell-penetrating, and antiviral tasks, CNN-based methods demonstrated higher performance than the approaches proposed in this work. However, these differences were not systematic or substantial.

Despite deep learning’s promising results, the further exploration of different architectures, hyperparameters, configuration strategies, and embedding representation evaluations is necessary to fully realize its potential. This is particularly important in separating antiviral peptides or peptides with communication and transmission properties. Moreover, applying transfer learning and fine-tuning approaches appears to be a promising alternative in addressing the challenges associated with low-N datasets. Future work should focus on these methods to enhance the model performance and generalization for properties like drug delivery, quorum sensing, and anti-aging peptide identification.

#### 2.2.3. Benchmark Analysis

This work explores various tools, methods, and strategies previously reported in the literature to facilitate a comparison of their performance with the trained models using the implemented pipeline and available on AMP-Detector.

First, we performed a literature survey to identify state-of-the-art sequence-based approaches for the prediction of the functional biological activity of peptide sequences. The search included methods with web servers, available models, or strategies for the training of classification models based on requirements outlined in public repositories and the available datasets. Subsequently, all collected tools were evaluated and tested, excluding those that were inaccessible (non-functional servers), lacked the necessary datasets, did not have auxiliary tools for numerical representation, or could not be replicated locally.

Using the collected tools, a benchmark analysis was performed on the benchmark dataset (the 10% of the input data that was excluded from the model development stage), assessing more than 15 tools previously reported in the literature. This analysis focused on commonly reported biological activity types, such as antimicrobial, antibacterial, and antiviral classifications. The sensitivity and specificity (see Section S1 of the Supplementary Materials) were calculated to evaluate and compare the performance of the explored tools and the trained models.

The results are summarized in Table 2. The trained model using the proposed pipeline in this work achieved the highest sensitivity and specificity for the antimicrobial task. The DBAASP *klebsiella* method achieved the highest sensitivity but had random specificity for antibacterial classification. DBAASP approaches depend on the types of organisms used to evaluate or detect antibacterial peptides, with methods like DBAASP *staphylococcus* showing sensitivity lower than 0.9, decreasing by more than 0.05 points. Tools like TPpred-LE [30] exhibited the highest sensitivity for antifungal, antiparasitic, and antiviral agents but generally had low specificity.

In summary, the models available in AMP-Detector achieved the highest specificity across all evaluated tasks, particularly excelling in antimicrobial identification. They also showed competitive performance in other tasks. The high specificity of the trained models facilitates their use in therapeutic peptide discovery, allowing for the effective elimination of peptides without desirable activity and increasing the likelihood of detecting peptides with the desired activity.

### 2.3. Case Study: Antimicrobial Peptide Discovery and De Novo Peptide Generation

**Figure 3:**
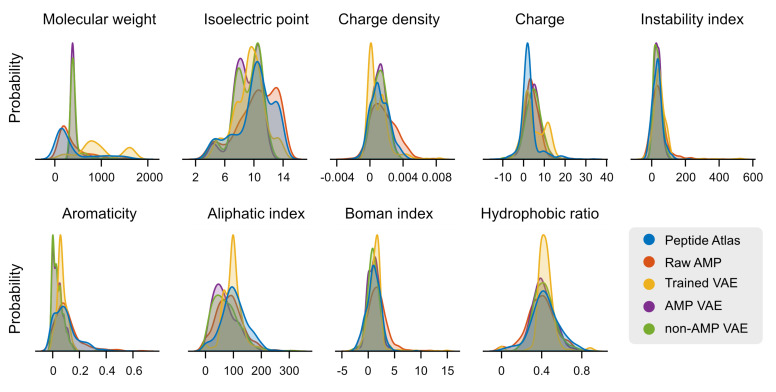
**Physicochemical property distribution analysis reveals concordance between existing and generated peptide sequences**. Using modlAMP, we explored the similarities between sequences of different sources for nine physicochemical properties. The de novo generated sequences showed differing distributions for the molecular weight, charge, and aromaticity; however, no significant differences were observed in other properties. This suggests that the models generated are reliable and produce sequences consistent with those previously reported. The Y-axes have been removed from the subplots for clarity; the values do not play any role when comparing the shapes of the probability distributions.

Here, we used AMP-Detector to discover and generate potential antimicrobial peptides. First, we extracted and applied our models on over 3.6 million peptides with unknown activity from the Peptide Atlas database [27]. We identified over 300,000 peptides with potential antimicrobial activity and 400,000 others with potential antibacterial and antifungal activity (see Table 3). We found that more than 30% of the peptides analyzed exhibited potential activity in antiviral, antidiabetic, blood–brain-barrier-penetrating, anti-inflammatory, quorum-sensing, and neuropeptide functions. This high prevalence of potential activity among the peptides analyzed is somewhat unusual and might be due to the high rate of false positives. To further explore the likelihood of these findings, we analyzed the distribution of the physicochemical properties using the modlAMP v4.3.0 tool [41] and how these differed among the datasets studied and generated. No differences were observed between the raw data and the peptides from the Peptide Atlas (see Figure 3), meaning that the activity identified was indeed feasible.

As a second example, we incorporated the models here developed with generative learning techniques for **de novo** peptide design. We explored two approaches: (i) emulating transfer learning strategies using a pre-trained model to generate metalloproteins [42] and (ii) training a generative model based on VAE architectures following the method proposed by [43]. Two scenarios were examined for the pre-trained model approach: (i) generating new sequences from peptides with reported antimicrobial activity in the literature and (ii) applying the same methodology to peptides without reported antimicrobial activity. Table 3 summarizes the peptides identified with the desired activity. In both scenarios, 100,000 peptides were generated and evaluated using the predictive models developed in this study. Table 3 shows no significant differences observed in the peptides identified for each activity type based on the input used. However, more peptides were consistently identified when using peptides with reported antimicrobial activity as input for de novo peptide generation. The physicochemical properties, such as the isoelectric point and hydrophobic radius, exhibited subtle differences in their distributions across the generated peptides (cf. Figure 3).

**Figure 4:**
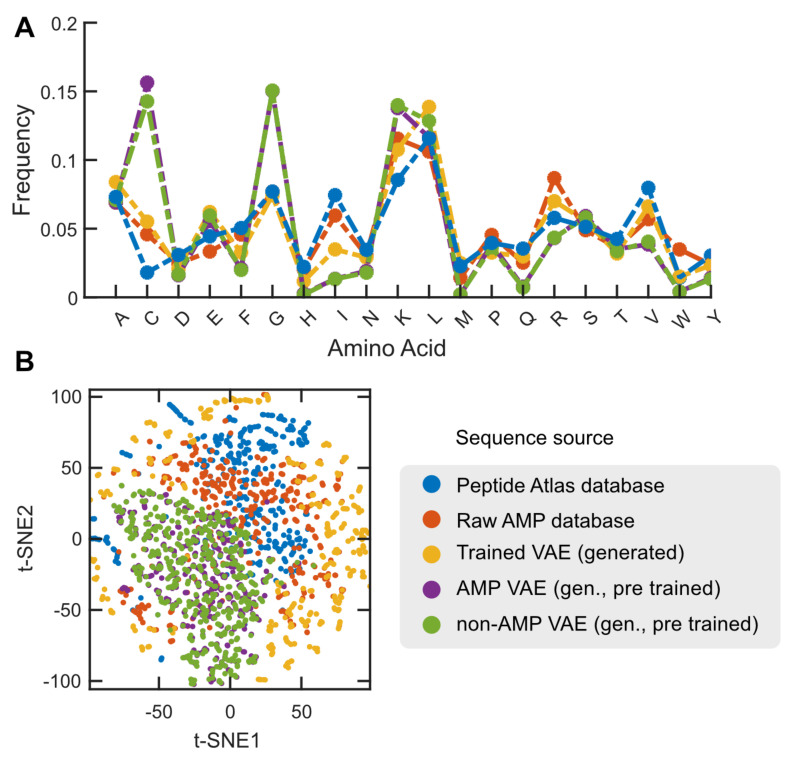
**Visualization of generated antimicrobial peptides by applying VAE approaches.** (**A**). Averagefrequency of amino acids in the studied sequences depends on their source of origin. Sequences created using pre-trained VAEs tend to have slightly more cysteine and glycine instances, regardless of whether the original input was an AMP or not. On the other hand, raw AMPs, potential AMPs identified in the Peptide Atlas, and AMPs generated using VAE trained with AMPs all show similar patterns, except for isoleucine and leucine. In these cases, the peptides generated using VAEs have a lower or higher frequency, respectively (see Table S4 in the Supplementary Materials for more details). (**B**). Embedding visualization via t-SNE for the numerical representations generated by the ProTrans t5 Uniref pre-trained model for the different sources analyzed. The sequences generated by the VAE trained with AMP sequences show greater dispersion and visual separation compared to other sources, indicating possible new behaviors. This is reflected in the variations in the amino acid properties and frequency. The representations for the potential AMPs generated via the pre-trained VAE exhibit similar behavior. The same is true for the raw AMP sequences and the potential AMPs identified in the Peptide Atlas, consistent with the analysis of the properties and amino acid frequencies.

We used our generative model to create 1000 new peptide sequences, but 355 were not unique and were discarded. Out of the remaining 645, we identified 640 with potential antimicrobial activity. This outcome aligns with the model’s training stage, which utilized antimicrobial peptide sequences to learn about amino acid frequencies and relationships, and is also reflected in the relative frequency of their constitutive amino acids (Figure 4A). Subsequent classification revealed that over 500 of these peptides could have antifungal and antiparasitic properties, while more than 300 may possess antibacterial and antimalarial properties.

To further explore the relationship between the physicochemical properties and models in the discovered sequences, we studied their distributions in different datasets. We randomly selected 600 peptides from each strategy and dataset, distinguishing between peptides registered in the Peptide Atlas, those generated using the VAE pre-trained models and our VAE model, and the raw AMP peptides. We used the modlAMP tool to estimate nine physicochemical properties of these sequences (cf. Figure 3 regarding how these properties are distributed among the datasets and Section S10 of the Supplementary Materials for more details). Our findings showed that the sequences generated using our VAE model had a higher molecular weight than those in other datasets, but no substantial differences in other properties were observed. This indicates that the generated models are stable and produce sequences that are consistent with those already reported.

Compared to other analyzed strategies, the differences observed in the peptides with potential antimicrobial activity, which were generated using the trained VAE, can be attributed to the trends in the residues present in the generated sequences. For instance, the higher occurrence of hydrophobic residues such as leucine and methionine in the peptide sequences indicates their increased hydrophobicity (see Figure 4A). Additionally, the lower frequency of charged residues affects properties like the charge density and isoelectric point.

## 3. Materials and Methods

### 3.1. Data Collection and Preprocessing

We obtained peptide sequences from the Peptipedia v2.0 [28], including only those with biological activity reported in the literature. Filters based on the target biological activity were implemented to collect positive examples. These positive examples included antimicrobial, antibacterial, antiviral, antifungal, antiparasitic, anuran defense, antiprotozoal, antiangiogenic, antidiabetes, antibacterial Gram(+) and Gram(−), anti-inflammatory, antimalarial, antimammalian cell, antioxidative, blood–brain-barrier-penetrating, cell–cell communication, cell-penetrating, drug delivery vehicles, neuropeptides, and quorum sensing, among others. For each dataset, negative examples were collected using peptide sequences in Peptipedia v2.0 with reported biological activity differing from the positive instances [28]. For example, all peptides without antimicrobial effects were used to generate negative examples for the antimicrobial dataset.

Using a binary dataset as input, the examples were divided by category (positive or negative). Then, the CD-Hit v4.8.1 tool [44] was applied to remove redundancies in each category using a homology percentage of 90% and the rest of the configuration parameters by default [33]. Finally, the representative sequences were employed to rebuild the binary classification dataset. We addressed the class imbalance undersampling issue by randomly selecting subsets of the negative class, as it was substantially larger than the positive class in all datasets.

### 3.2. Encoding Peptide Sequences

Encoding, i.e., the numerical representation of the peptide sequences, is one critical task when developing predictive models in protein science. Here, we explored different pre-trained models (summarized in Table 4). The bio-embedding [45] tool was used to apply the pre-trained models and generate the embeddings, combined with its reduced dimensionality method. We also explored classic numerical representation strategies, including one-hot encoding [46], physicochemical coding [47], and Fast Fourier Transform approaches [48], to evaluate the performance of the machine learning algorithms using different numerical representation strategies.

### 3.3. Training and Validating Classification Models

The classification models developed in this work were built based on a classic pipeline used to train predictive models using machine learning approaches [50]. Figure 1 summarizes this work’s proposed method for the training of sequence-based predictive models.

First, the dataset is encoded as described in the previous section. Subsequently, each encoded dataset is split into two datasets in a 90:10 ratio. The first dataset is used for model development and the second for independent testing and benchmarking (see Figure 1A).

The model development dataset is divided into training and validation datasets using an 80:20 ratio. Within this stage, we develop models using diverse supervised learning algorithms and hyperparameters. The supervised learning algorithms explored in this work include tree-based algorithms like decision trees; ensemble-based methods like ExtraTrees, Hist Gradient Boosting, XGBoost, and Random Forest; distance-based algorithms like *K*-Nearest Neighbors; and kernel-based methods like Support Vector Machine. Each model is evaluated using classic performance metrics such as precision, recall, accuracy, the F-score, and the Matthews correlation coefficient (MCC) (for details, see Section S1, Supplementary Materials). A *k*-fold cross-validation with k=10 is employed to prevent overfitting. We repeat the whole process (i.e., partitioning into training and validation datasets and exploring various algorithmic combinations, hyperparameters, and numerical representations) 30 times to generate distributions of the performance metrics and obtain robust results (see Figure 1B). Subsequently, we identify the best combinations of numerical representation strategies and supervised learning algorithms by using the methods proposed in [26] (see Figure 1C and Section S2 in the Supplementary Materials for more details). Lastly, the selected combinations of machine learning algorithms and encoding strategies are used as input for a Bayesian hyperparameter calibration process [51] (see Figure 1C).

We utilize the independent testing dataset to assess the performance of the models with the optimized hyperparameters produced in the previous stage. Performance comparisons between the validation and testing stages serve as tools for the selection of the best model, considering factors such as (i) the highest performance in both the testing and validation sets, (ii) minimal differences between the validation and testing performance, and (iii) low overfitting rates during training. Finally, the models are saved and exported with the chosen combination of numerical representation strategies, supervised learning algorithms, and optimized hyperparameters to further explore new, unknown peptides (see Figure 1D).

### 3.4. Benchmark Analysis

The performance obtained using the proposed methodology to train the binary classification models was compared with that of tools, libraries, and predictive models previously reported in the literature (cf. Section S3 in the Supplementary Materials for more details). We conducted a literature survey on reported classification models regarding antimicrobial peptide activity, including the subtypes analyzed in this work: antibacterial, antiviral, antifungal, and antiparasitic. Then, we evaluated the feasibility of using each tool, following the instructions described in the user manual, README, or descriptions on each repository or web platform. All tools that could be tested and used correctly were selected and applied to the benchmark dataset, obtaining the classifications of each peptide evaluated. The performance of each tested tool was assessed using classic performance metrics.

### 3.5. Discovering Potential Peptides with Desirable Biological Activity from the Peptide Atlas

All peptide sequences from the Peptide Atlas [27] were collected and processed using filters of the same length and a canonical residue evaluation and encoded using the method associated with the best-performing models determined in this work. The models give a probability for each category (has activity or has no activity) and are combined with a threshold value to prevent error predictions (false positive or false negative predictions). In this case, the threshold applied was estimated using the AUC score (see Table S4, Supplementary Materials, for more details).

### 3.6. De Novo Design of Antimicrobial Peptides Using VAE

This work applied two VAE strategies to explore generative approaches for the generation of de novo potential antimicrobial peptides. First, we generated 100,000 novel peptide sequences using the previously collected antimicrobial peptide dataset and the model implemented by Greener et al. [42]. We analyzed the resulting dataset to remove redundancies and exclude results already reported in Peptipedia [28] and the Peptide Atlas [27].

The second strategy is based on the architecture and methods proposed by Hawkins-Hooker et al. [43]. Using the processed antimicrobial peptide dataset, a VAE model is trained by applying the architecture proposed in [43]. Then, 100,000 novel peptide sequences are generated using the trained models and the antimicrobial peptide dataset. The same filters are applied to discard redundancies and coincidences with the Peptipedia and Peptide Atlas databases.

Once the novel peptide sequences are generated, we apply the models and encoding strategies developed in this work to classify these unknown peptide sequences. The stages are (i) applying numerical representation for each classification model and (ii) predicting the novel sequences using the antimicrobial classification model and the different subtypes of classification models, like antiviral, antibacterial, and anuran defense. All classification models use a threshold to generate the classification based on the probability predicted for each category type (has activity or has no activity) on each model. This work applies a threshold of 0.7 to reduce the probability of errors in classification.

Finally, the classified peptides are explored based on their moonlighting properties and compared with the reported antimicrobial peptides and the predictions of novel potential antimicrobial peptides detected from the Peptide Atlas database.

### 3.7. Implementation Strategies

All source code, including the modules, libraries, and demonstration scripts in the built library, was implemented in Python v3.9.16. The classification models were implemented using the packages available in the DMAKit v1.0.0 library [46]. The Bayesian hyperparameter optimization strategies were developed using the Optuna library [51]. Finally, a conda environment was constructed to facilitate the deployment of the constructed library, combined with different Jupyter Notebooks, to ensure the replicability of the presented work. AMP-Detector was implemented as an executable tool to facilitate the inference and exploration of unknown peptide sequences. It accepts a Fasta format file and allows the evaluation of one or multiple types of activity. AMP-Detector processes the input arguments, including the peptide sequences and the activity to be evaluated. Next, it applies the embedding representation and loads the classification models, which are then used to predict the associated biological activity. The tool generates a CSV file as output, containing the sequences and all predictions produced by the classification models. All source code and environmental configurations are available under the MIT licence for non-commercial use in the GitHub repository https://github.com/ProteinEngineering-PESB2/amp_class_ml. Moreover, all raw and coded datasets, the trained models exported in joblib format, the discovered and explored peptide sequences from the Peptide Atlas database, and the sequences generated through generative learning using VAEs are publicly available for non-commercial use at the Google Drive link https://drive.google.com/drive/folders/1IO_mL6Jt7vGQZ6aE7lK6crQFiLzZ62Cf?usp=sharing.

## 4. Conclusions

The novel contributions of our work are threefold. Firstly, we developed a sequence-based approach to create functional classification models by combining protein language models and classical machine learning methods. Secondly, we used this approach to build AMP-Detector, a Python library integrated with 21 models for the classification of various biological activity types, such as antimicrobial, antibacterial, and antiviral, achieving an average precision of over 83%. The comparative analysis demonstrated that AMP-Detector exhibits higher specificity and sensitivity compared to other state-of-the-art methods. Lastly, we assessed the performance of these models in identifying and generating new peptides with potential biological activity. We identified more than 300,000 potential AMPs in the Peptide Atlas and proposed over 100,000 new sequences using a pre-trained VAE in a transfer learning-inspired scheme and a new VAE trained on AMP sequences.

The combination of classification model design strategies, trained models, and integration strategies in design and discovery methods for potential AMPs demonstrates the benefits of ML-based methods in expediting the discovery of peptides with pharmaceutical activity. This approach also helps in designing de novo therapeutic peptides and represents a competitive and widely applicable strategy for the study of peptides with specialized biological activity, such as anticancer, antiviral, or antibacterial properties.

Our future work will consider the development of evaluation models for pharmacological properties, such as the half-life and IC50, and assess adverse effects like toxicity, cytotoxicity, immunogenicity, and allergic effects. Additionally, we will explore strategies like generative adversarial networks and diffusion models for the creation of new peptides and then compare these strategies to determine the most effective approach. We also intend to investigate methodological components related to designing classification models for underrepresented biological activity types, utilizing transfer learning and semi-supervised learning techniques to develop efficient and generalized classification models. These efforts are aimed at facilitating the autonomous design of peptides with desirable therapeutic properties through integrative ML methods.

## Figures and Tables

**Table 1:** Precision for selected models for each evaluated task, considering all stages of the training process and available on AMP-Detector.

Task	Algorithm	Encoder	Training Performance	Validation Performance	Testing Performance
Antiangiogenic	HistGradientBoosting	ProTrans t5 BDF	(0.75, 0.78)	(0.74, 0.76)	0.82
Antidiabetic	RandomForest	ProTrans t5 Uniref	(0.81, 0.83)	(0.81, 0.82)	0.81
Anti-Gram (−)	XGBClassifier	Esm1B	(0.89, 0.89)	(0.88, 0.89)	0.88
Anti-Gram (+)	ExtraTrees	Esm1B	(0.88, 0.88)	(0.88, 0.88)	0.88
Anti-inflammatory	Random Forest	ProTrans t5 xlu50	(0.87, 0.88)	(0.87, 0.87)	0.89
Antimalarial	ExtraTrees	ProTrans t5 BERT	(0.82, 0.85)	(0.83, 0.84)	0.78
Antimammalian cell	ExtraTrees	ProTrans t5 Uniref	(0.90, 0.90)	(0.90, 0.90)	0.90
*Anti-methicillin-resistant S. aureus*	ExtraTrees	Esm1B	(0.87, 0.88)	(0.87, 0.87)	0.90
Antioxidative	RandomForest	ProTrans t5 xlu50	(0.75, 0.77)	(0.75, 0.76)	0.82
Antibacterial	RandomForest	ProTrans t5 xlu50	(0.89, 0.90)	(0.89, 0.89)	0.92
Antifungal	RandomForest	ProTrans t5 xlu50	(0.83, 0.83)	(0.82, 0.82)	0.84
Antimicrobial	RandomForest	ProTrans t5 Uniref	(0.88, 0.88)	(0.87, 0.88)	0.88
Antiparasitic	RandomForest	Esm1b	(0.83, 0.84)	(0.83, 0.83)	0.85
Antiviral	RandomForest	ProTrans t5 Uniref	(0.81, 0.81)	(0.80, 0.81)	0.79
Anuran defense	Hist Gradient Boosting	ProTrans t5 xlu50	(0.90, 0.91)	(0.90, 0.90)	0.93
Blood–brain barrier penetrating	ExtraTrees	ProTrans t5 Uniref	(0.77, 0.80)	(0.78, 0.79)	0.85
Cell–cell communication	Hist Gradient Boosting	ProTrans t5 xlu50	(0.90, 0.91)	(0.90, 0.90)	0.91
Cell-penetrating	ExtraTrees	ProTrans t5 ALBERT	(0.87, 0.88)	(0.87, 0.87)	0.86
Neuropeptide	ExtraTrees	ProTrans t5 xlu50	(0.86, 0.86)	(0.86, 0.86)	0.87
Quorum sensing	ExtraTrees	ProTrans t5 ALBERT	(0.83, 0.86)	(0.87, 0.88)	0.87
Drug delivery vehicle	ExtraTrees	Esm1B	(0.85, 0.85)	(0.84, 0.85)	0.92

**Table 2:** Benchmarking of our models with state-of-the-art methods reported in the literature (In bold, the highest performance for each evaluated metric and for each method in all evaluated tasks).

Task	Method	Reference	Sensitivity	Specificity	F1
Antimicrobial	**AMP-Detector**	—	**0.91**	**0.85**	**0.88**
	AMP-discover	[31]	0.66	0.78	0.74
	amplify	[32]	0.8	0.7	0.72
	TPpred-LE	[30]	0.62	0.71	0.71
	AMPScanner	[33]	0.59	0.55	0.55
Antifungal	**AMP-Detector**	—	**0.85**	**0.79**	**0.84**
	AMPfun	[34]	0.6	0.56	0.67
	IAMP-RAAC	[35]	0.59	0.54	0.67
	AMP-discover	[31]	0.53	0.38	0.61
	DeepAFP	[36]	0.55	0.44	0.52
	TPpred-LE	[30]	0.75	0.46	0.14
Antibacterial	**AMP-Detector**	—	0.95	**0.81**	**0.96**
	AMP-discover	[31]	0.92	0.39	0.89
	**AntiBP3 Gram variable**	[37]	**0.98**	0.39	0.86
	AntiBP3 Gram (−)	[37]	0.94	0.3	0.82
	AntiBP3 Gram +	[37]	0.94	0.3	0.82
	**DBAASP** *** E. coli***	[38]	**0.98**	0.21	0.59
	**DBAASP** *** S. aureus***	[38]	**0.98**	0.19	0.49
	AMPActiPred	[38]	0.84	0.13	0.49
	DBAASP Klebsiella	[38]	0.99	0.18	0.45
	TPpred-LE	[30]	0.97	0.17	0.41
	DBAASP Pseudomonas	[38]	0.97	0.17	0.4
Antiparasitic	**AMP-Detector**	—	0.89	**0.8**	**0.84**
	AMP-discover	[31]	0.56	0.56	0.66
	AMPfun	[34]	0.67	0.48	0.2
	IAMP-RAAC	[35]	0.49	0.46	0.16
	multipep_max	[39]	0.71	0.47	0.07
	TPpred-LE	[30]	**1.0**	0.47	0.01
Antiviral	**AMP-Detector**	—	0.76	**0.77**	**0.78**
	IAMP-RAAC	[35]	0.72	0.65	0.68
	**TPpred-LE**	[30]	**0.89**	0.65	0.67
	AMPfun	[34]	0.75	0.63	0.66
	AMP-discover	[31]	0.56	0.57	0.66
	DeepAVP	[40]	0.52	0.46	0.57
	AVP-IFT	[12]	0.52	0.47	0.46

**Table 3:** Integrating AMP-Detector to evaluate unknown peptide sequences obtained from non-annotated databases or de novo-generated peptides using generative learning methods.

Activity	# Discovered from Peptide Atlas	# Generated Using Trained VAE	# Generated Using Positive Examples	# Generated Used Negative Examples
Antibacterial	403,367	336	63,709	58,826
Anti-Gram (+)	83,271	147	34,468	31,960
Antifungal	406,191	554	37,147	37,976
Blood–brain barrier penetrating	1,259,618	38	12,384	11,277
Antiparasitic	133,555	563	2887	2077
Anti-inflamatory	2,999,776	1	27	27
Cell-penetrating	698,536	58	12,468	14,066
Anti mammalian cell	81,701	93	16,360	14,611
Anuran defense	593,692	40	4729	4554
*Anti-methicillin-resistant S. aureus*	29,964	114	12,984	12,774
Cell–cell communication	129,651	2	13,237	13,475
Antioxidative	290,289	75	5307	4821
Antiangiogenic	292,815	34	37,954	33,980
Antiviral	2,582,176	29	24,987	24,178
Quorum sensing	2,088,671	17	16,477	15,624
Antimicrobial	305,496	640	49,497	45,325
Antimalarial	259,169	311	35,862	34,647
Anti-Gram (−)	152,568	232	37,009	33,168
Drug delivery vehicle	113,0481	60	14,147	15,857
Antidiabetic	3,344,580	1	4571	4518
Neuropeptide	2,499,312	1	20,188	19,898

**Table 4:** Summary of pre-trained models employed for numerical representation of input dataset.

#	Pre-Trained Model	Description	Tensor Size	Reference
1	ProTrans t5 UniRef	The ProtTrans UniRef pre-trained model is a deep learning model specifically trained for protein sequence representation and understanding. It is trained on the UniRef50 database, which contains clustered protein sequences to reduce redundancy and improve diversity.	1024	[49]
2	ProTrans t5 xlu50	ProtT5-XL-UniRef50 is based on the t5-3b model and was pre-trained on a large corpus of protein sequences in a self-supervised fashion. This means that it was pre-trained on the raw protein sequences only, with no humans labeling them in any way (which is why it can be used with a large amount of publicly available data), with an automatic process to generate inputs and labels from the protein sequences.	1024	[49]
3	ProTrans T5-BDF	ProtT5-XL-BFD is based on the t5-3b model and was trained on a large corpus of protein sequences in a self-supervised fashion. This means that it was trained on the raw protein sequences only, with no human labeling them in any way (which is why it can use many publicly available data), with an automatic process to generate inputs and labels from the protein sequences.	1024	[49]
4	Esm1b	The ESM-1b (Evolutionary Scale Modeling) pre-trained model is a variant of the ESM model, designed for protein sequence modeling. It is based on self-supervised learning techniques and utilizes a Transformer architecture, similar to those used in natural language processing tasks.	1280	[45]
5	ProTrans XLNet	The ProtTrans XLNet pre-trained model is a variant of the XLNet model customized for protein sequence analysis. XLNet is an extension of the Transformer-based architecture, which integrates bidirectional context learning with permutation-based training. Similarly, ProtTrans XLNet leverages these features to learn contextual representations of amino acids in protein sequences.	1024	[49]
6	ProTrans ALBERT	The ProtTrans ALBERT (A Lite BERT) pre-trained model is a variant of the ALBERT model specifically adapted for protein sequence analysis. ALBERT is a lightweight version of the BERT model, designed to reduce the computational resource usage while maintaining its performance. Similarly, ProtTrans ALBERT leverages this efficiency to provide effective representations of amino acids in protein sequences.	4096	[49]
7	ProTrans BERT	The ProtTrans BERT (Bidirectional Encoder Representations from Transformers) pre-trained model is a variant of the BERT model specifically tailored to protein sequence analysis. Like its counterpart in natural language processing, ProtTrans BERT utilizes a Transformer-based architecture to learn contextual representations of amino acids in protein sequences.	1024	[49]

## Data Availability

The source code, example dataset, obtained results, and trained models joined with different Jupyter Notebooks and an environment config file are available at the GitHub repository https://github.com/ProteinEngineering-PESB2/amp_class_ml. The Supplementary Materials summarize the source code and all applications used to run and explore the generative approaches with their respective link access. The Google Drive link https://drive.google.com/drive/u/1/folders/1IO_mL6Jt7vGQZ6aE7lK6crQFiLzZ62Cf provides all raw data used in this work, the trained model in *.joblib format, the exploration and tuning hyperparameter results, and the generated and discovered potential antimicrobial peptides.

## Supplementary Materials

The following supporting information can be downloaded at https://www.mdpi.com/1422-0067/25/16/8851/s1. References [52,53,54,55,56,57,58,59,60,61,62,63,64,65,66,67,68,69,70,71,72,73,74,75,76,77,78,79,80,81,82,83] are cited in the supplementary materials.
