# Supplementary material for: Protein Language Models and Machine Learning Facilitate the Identification of Antimicrobial Peptides
Source: Int J Mol Sci. 2024 Aug 14;25(16):8851. doi: 10.3390/ijms25168851 (PMC11487388; doi:10.3390/ijms25168851)
Supplement: Supplementary file 1 [file ijms-25-08851-s001.zip › ijms-3089892-supplementary.pdf]

Supplementary Information: Protein language models and machine learning facilitate the identification of antimicrobial peptides

David Medina-Ortiz[1,2*], **Seba Contreras**[3†], **Diego Fernández**[1], **Nicole Soto-García**[1], **Iván Moya**[1], **Gabriel Cabas-Mora**[1], and **Álvaro Olivera-Nappa**[4]

[1]Departamento de Ingeniería En Computación, Universidad de Magallanes, Avenida Bulnes 01855, Punta Arenas, Chile.
[2]Departamento de Ingeniería Química, Biotecnología y Materiales, Universidad de Chile, Avenida Beauchef 851, Santiago, Chile.
[3]Max Planck Institute for Dynamics and Self-Organization, Am Faßberg 17, 37077 Göttingen, Germany
[4]Centre for Biotechnology and Bioengineering, CeBiB, Universidad de Chile, Beauchef 851, Santiago, Chile

S1 Metrics employed for evaluating performance of trained models

Let TP be the true positive values (correctly classified by the model), FP be the false positive (unexpected results), the FN the false negative (missing result), and the TN are the true negative (correctly absent of result). Using the defined ratios, it is possible to determine the more common metrics employed to evaluate the performance of a predictive model. The equations 1, 2, 3, 4, and 5 represent the classic metrics employed to evaluate the performance of the predictive models for classification tasks and the metrics utilised in this work. Besides, sensitivity and specificity were also used in this work, estimated by employing the equations 6 and 7, respectively.

$$accuracy = \frac{TP + TN}{TP + FP + TN + FN} \tag{S1}$$

$$precision = \frac{TP}{TP + FP} \tag{S2}$$

$$recall = \frac{TP}{TP + FN} \tag{S3}$$

$$F_\beta = \frac{(1+\beta^2)TP}{(1+\beta^2)TP + FP + \beta^2 FN} \tag{S4}$$

$$MCC = \frac{(TP \times TN) - (FP \times FN)}{\sqrt{(TP + FP)(TP + FN)(TN + FP)(TN + FN)}} \tag{S5}$$

$$Sensitivity = \frac{TP}{TP + FN} \tag{S6}$$

$$Specificity = \frac{TN}{TN + FP} \tag{S7}$$

*david.medina@umag.cl
†seba.contreras@ds.mpg.de

S2 Criteria for selecting best combinations of machine learning algorithm and numerical representation strategies

Let M be a metric to evaluate the performance of a binary classification model, and let $S\{M\}$ be a sample of performances related to the same algorithm and the same supervised learning algorithm but explored with different hyperparameters.

Each M was utilized to evaluate the training and validation stages of generating classification models.

Classic statistics like average and standard deviation were calculated for all distributions.

With the metrics accuracy, precision, recall, and F1-score declared in equations 1, 2, 3, and 4 a total of 16 assesses could be associated including:

- 4 evaluations related with the average on training stage.
- 4 evaluations related with the average on validation stage.
- 4 evaluations related with the standard deviation on training stage.
- 4 evaluations related to the standard deviation in the validation stage.

Using this point, a Bernoulli event could be evaluated by asking the following points:

- If a value is higher than the average for a metric M
- If a value is lower than the standard deviation for a metric M

Based on the 16 Bernoulli events, a random variable X could be defined. It has a binomial distribution with params $n = 16$ and $p = 0.5$. We have defined the success probability $P(X = 1) = 0.5$ to maintain the balance between a success and failure probability value.

Finally, using the generated distribution, a threshold t could filter the combinations of supervised learning algorithm and numerical representation strategy. In this case, a filter criterion was $P(X \geq 14)$ as suggested in (23). Alternatively, if the recommended number is not achieved, the highest voting value is employed.

S3 Previously reported methods, tools, and libraries explored to address the benchmark analysis

S3.1 Antibacterial peptide detection

- AMPActiPred
  - **Reference**: (43)
  - **Input**: Peptide sequence
  - **Status**: It works
- Diff-AMP
  - **Reference**: (36)
  - **Input**: Peptide sequence
  - **Status**: It works
- AntiBP3
  - **Reference**: (2)
  - **Input**: Peptide sequence
  - **Status**: It works
- StaBle-ABPpred
  - **Reference**: (31)
  - **Input**: Peptide sequence
  - **Status**: Not working
- Deep-ABPpred

  - **Reference**: (28)
  - **Input**: Peptide sequence
  - **Status**: Not working

- lstm_peptides
  - **Reference**: (45)
  - **Input**: Peptide sequence
  - **Status**: Not working

- AntiBP2
  - **Reference**: (16)
  - **Input**: Peptide sequence
  - **Status**: It works

S3.2 Antimicrobial peptide detection

- E-CLEAP
  - **Reference**: (37)
  - **Input**: Peptide sequence
  - **Status**: Not working

- SAMP
  - **Reference**: (9)
  - **Input**: Peptide sequence
  - **Status**: Not working

- AMPlify
  - **Reference**: (18)
  - **Input**: Peptide sequence
  - **Status**: It works

- iAMP-Attenpred
  - **Reference**: (40)
  - **Input**: Peptide sequence
  - **Status**: Not working

- AMP-BERT
  - **Reference**: (17)
  - **Input**: Peptide sequence
  - **Status**: It works

- iAMP-CA2L
  - **Reference**: (38)
  - **Input**: Peptide sequence
  - **Status**: Not working

- AmpGram
  - **Reference**: (3)
  - **Input**: Peptide sequence
  - **Status**: It works

- Deep-AmPEP30
  - **Reference**: (41)

  - **Input**: Peptide sequence
  - **Status**: Not working

- IAMPE
  - **Reference**: (15)
  - **Input**: Peptide sequence
  - **Status**: Not working

- AMAP
  - **Reference**: (13)
  - **Input**: Peptide sequence
  - **Status**: Not working

- AMPScanner
  - **Reference**: (34)
  - **Input**: Peptide sequence
  - **Status**: It works

- AMPPRED
  - **Reference**: (24)
  - **Input**: Peptide sequence
  - **Status**: Not working

- MLAMP
  - **Reference**: (21)
  - **Input**: Peptide sequence
  - **Status**: Not working

- Binary model
  - **Reference**: (27)
  - **Input**: Peptide sequence
  - **Status**: Not working

- iAMP-2L
  - **Reference**: (39)
  - **Input**: Peptide sequence
  - **Status**: Not working

- ClassAMP
  - **Reference**: (14)
  - **Input**: Peptide sequence
  - **Status**: Not working

- AMP
  - **Reference**: (35)
  - **Input**: Peptide sequence
  - **Status**: Not working

- AMPA
  - **Reference**: (33)
  - **Input**: Protein sequence
  - **Status**: It works

S3.3 Antiviral peptide detection

- AVP-IFT
  - **Reference**: (12)
  - **Input**: Peptide sequence
  - **Status**: It works
- AI4AVP
  - **Reference**: (20)
  - **Input**: Peptide sequence
  - **Status**: It works
- DeepAVP
  - **Reference**: (19)
  - **Input**: Peptide sequence
  - **Status**: It works
- FIRM-AVP
  - **Reference**: (5)
  - **Input**: Peptide sequence
  - **Status**: Not working
- AVPpred
  - **Reference**: (32)
  - **Input**: Peptide sequence
  - **Status**: Not working

S3.4 Antifungal peptide detection

- DeepAFP
  - **Reference**: (44)
  - **Input**: Peptide sequence
  - **Status**: It works
- Antifungal
  - **Reference**: (46)
  - **Input**: Peptide sequence
  - **Status**: Not working
- Deep-AFPpred
  - **Reference**: (29)
  - **Input**: Peptide sequence
  - **Status**: Not working
- Antifp
  - **Reference**: (1)
  - **Input**: Peptide sequence
  - **Status**: It works

S3.5 Antiparasitic peptide detection

- PredAPP
  - **Reference**: (Zhang et al.)
  - **Input**: Peptide sequence
  - **Status**: Not working

S3.6 Multi task peptide detection

- TPpred-LE
  - **Reference**: (22)
  - **Input**: Peptide sequence
  - **Task**: antimicrobial, toxic, antibacterial, antiinflammatory, antiviral, anticancer, antifungal, drug delivery vehicle, cell-penetrating, cell–cell communication, antiparasitic, antiangiogenic, antihypertensive peptide), polystyrene surface-binding, and quorum sensing peptides
  - **Status**: It works

- UniDL4BioPep
  - **Reference**: (8)
  - **Input**: Peptide sequence
  - **Task**: antibacterial, anti-hypertension, antidiabetes, bitter, umami, antimicrobial, antimalarial, anticancer, anti-methicillin-resistant S. aureus (MRSA) strains, tumor T cell antigens, blood–brain barrier, antiparasitic, neuropeptide, antifungal, antiviral, toxicity and antioxidant peptides
  - **Status**: It works

- PrMFTP
  - **Reference**: (42)
  - **Input**: Peptide sequence
  - **Task**: antibacterial, ACP, anticoronavirus, anti-diabetic, antiendotoxin, antifungal, antiHIV peptide, antihypertensive, antiinflammatory, antiMRSA, antiparasitic, antitubercular, antiviral, blood-brain barrier, biofilm-inhibitory, chemotactic, cell-penetrating, dipeptidyl peptidase IV peptide, quorum-sensing, surface-binding and THP peptides
  - **Status**: Not working

- MultiPep
  - **Reference**: (11)
  - **Input**: Peptide sequence
  - **Task**: antimicrobial, antivirus, antiparasitic, anticancer, antibacterial, antifungal, neuropeptide, drug administration, antihypertensive, toxic and antidiabetic peptides
  - **Status**: It works

- IAMP-RAAC
  - **Reference**: (7)
  - **Input**: Peptide sequence
  - **Task**: Atimicrobial, antiparasitic, antifungal, targeting gram-negative, targeting gram-positive, anticancer, antiviral and targeting mammals peptides
  - **Status**: It works

- AMP-discover
  - **Reference**: (25)
  - **Input**: Peptide sequence
  - **Task**: antimicrobial, antibacterial, antifungal, antiparasitic and antiviral peptides
  - **Status**: It works

- AMPfun
  - **Reference**: (6)
  - **Input**: Peptide sequence
  - **Task**: antiparasitic, antiviral, antifungal and anticancer peptides
  - **Status**: It works

- DBAASP v3
  - **Reference**: (26)
  - **Input**: Peptide sequence
  - **Task**: antibacterial, antifungal and antiviral peptides
  - **Status**: It works
- PepBio
  - **Reference**: (30)
  - **Input**: Peptide sequence
  - **Task**: antibacterial, anticancer, antifungal and antiviral peptides
  - **Status**: Not working

S4 Numerical representation strategies

This work explores different numerical representation strategies to encode peptides as numerical vectors. Table **S1** summarizes the numerical representation strategies used in this work and the tensor size generated for each one.

Supplementary Table S1: Numerical representation strategies and generated tensor size explored in this work

#	Numerical representation strategy	Tensor size
1	One Hot encoding	3,000
2	Physicochemical properties	150
3	FFT-based physicochemical properties	75
4	ProTrans t5 Uniref	1024
5	Esm1B	1280
6	ProTrans ALBERT	4096
7	ProTrans t5 xlu50	1024
8	ProTrans T5-BDF	1024
9	ProTrans XLNet	1024
10	ProTrans BERT	1024

S5 Summary of selected strategies for tuning hyperparameter process

Table **S2** summarizes the selected combinations for each explored task, considering the supervised learning algorithm, the numerical representation strategy, and the obtained voting.

Supplementary Table S2: Summary of selected combinations for tuning hyperparameters process for each evaluated task

Task	Algorithm	Encoder	Voting
	ExtraTrees	Esm1B	16
	ExtraTrees	ProTrans t5 BERT	16
Anti_malarial	ExtraTrees	ProtTrans t5 Uniref	16
	ExtraTrees	ProTrans t5-xlu50	16
	RandomForest	ProTrans t5-xlu50	16
Anti oxidative	RandomForest	ProtTrans t5 Uniref	16
	RandomForest	ProTrans t5-xlu50	16
	ExtraTrees	Esm1B	16
	ExtraTrees	ProtTrans t5 Uniref	16
	ExtraTrees	ProTrans t5-xlu50	16
Antimicrobial	Hist Gradient Boosting	ProTrans t5-xlu50	16

	RandomForest	ProtTrans t5 Uniref	16
	XGBoost	ProtTrans t5 Uniref	16
	XGBoost	ProTrans t5-xlu50	16
Antibacterial	ExtraTrees	ProTrans t5-xlu50	16
	RandomForest	ProTrans t5-xlu50	16
	XGBoost	ProTrans t5-BDF	14
Anuran defense	ExtraTrees	one_hot	16
	ExtraTrees	ProtTrans t5 Uniref	16
	ExtraTrees	ProTrans t5-xlu50	16
	Hist Gradient Boosting	ProTrans t5-xlu50	16
Anti methicillin-resistant *S. aureus*	ExtraTrees	Esm1B	16
	RandomForest	Esm1B	16
	RandomForest	ProtTrans t5 Uniref	16
	RandomForest	ProTrans t5-xlu50	16
Antifungal	ExtraTrees	Esm1B	12
	ExtraTrees	ProtTrans t5 Uniref	12
	ExtraTrees	ProTrans t5-xlu50	12
	Hist Gradient Boosting	Esm1B	12
	Hist Gradient Boosting	ProtTrans t5 Uniref	12
	RandomForest	Esm1B	12
	XGBoost	Esm1B	12
	XGBoost	ProTrans t5-BDF	12
	XGBoost	ProtTrans t5 Uniref	12
Cell penetrating	ExtraTrees	ProTrans t5 ALBERT	16
Antiparasitic	ExtraTrees	ProTrans t5-xlu50	16
	RandomForest	ProTrans t5-xlu50	16
Anti inflammatory	ExtraTrees	ProtTrans t5 Uniref	8
	Hist Gradient Boosting	ProtTrans t5 Uniref	8
	Hist Gradient Boosting	ProTrans t5-xlu50	8
	RandomForest	ProtTrans t5 Uniref	8
	RandomForest	ProTrans t5-xlu50	8
	XGBoost	ProtTrans t5 Uniref	8
	XGBoost	ProTrans t5-xlu50	8
Anti gram -	ExtraTrees	Esm1B	16
	ExtraTrees	ProTrans t5-xlu50	16
	Hist Gradient Boosting	Esm1B	16
	RandomForest	Esm1B	16
	XGBoost	Esm1B	16
Antiviral	ExtraTrees	Esm1B	16
	Hist Gradient Boosting	ProTrans t5-xlu50	16
	RandomForest	Esm1B	16
	RandomForest	ProtTrans t5 Uniref	16
	RandomForest	ProTrans t5-xlu50	16
	XGBoost	ProTrans t5-BDF	16
	XGBoost	ProtTrans t5 Uniref	16
Drug delivery vehicle	ExtraTrees	Esm1B	16
	ExtraTrees	ProTrans t5 ALBERT	16
	ExtraTrees	ProTrans t5-BDF	16
	ExtraTrees	ProtTrans t5 Uniref	16
	Hist Gradient Boosting	Esm1B	16
	RandomForest	ProtTrans t5 Uniref	16
Anti mammalian cell	Hist Gradient Boosting	ProtTrans t5 Uniref	16
Anti angiogenic	Hist Gradient Boosting	ProTrans t5-BDF	16

	RandomForest	Esm1B	16
Blood-brain barrier penetrating	ExtraTrees	ProtTrans t5 Uniref	13
Cell-cell communication	ExtraTrees	ProTrans t5-xlu50	16
	Hist Gradient Boosting	ProtTrans t5 Uniref	16
	Hist Gradient Boosting	ProTrans t5-xlu50	16
	RandomForest	ProtTrans t5 Uniref	16
Neuropeptide	ExtraTrees	Esm1B	16
	ExtraTrees	ProtTrans t5 Uniref	16
	ExtraTrees	ProTrans t5-xlu50	16
Anti diabetic	ExtraTrees	ProTrans t5-BDF	16
	ExtraTrees	ProtTrans t5 Uniref	16
	Hist Gradient Boosting	ProtTrans t5 Uniref	16
	RandomForest	ProTrans t5-BDF	16
	RandomForest	ProtTrans t5 Uniref	16
Anti gram +	ExtraTrees	Esm1B	12
	ExtraTrees	ProtTrans t5 Uniref	12
	Hist Gradient Boosting	ProTrans t5-xlu50	12
	XGBoost	ProtTrans t5 Uniref	12
	XGBoost	ProTrans t5-xlu50	12
Quorum sensing	ExtraTrees	ProTrans t5 ALBERT	16
	ExtraTrees	ProTrans t5 BERT	16
	ExtraTrees	ProTrans t5-xlu50	16
	RandomForest	Esm1B	16
	RandomForest	ProTrans t5 ALBERT	16

S6 Performances for trained and selected models in this work

Table **S3** summarizes the performances of the selected models trained in this work. Five metrics are considered, including all metrics described in section S1.

Supplementary Table S3: Summary of testing performances for selected models

Configuration	Activity	Accuracy	F1	Precision	Recall	MCC
ProTrans T5-BDF HistGradientBoosting	Anti angiogenic	0.824	0.824	0.824	0.824	0.636
ProTrans t5 Uniref RandomForest	Anti diabetic	0.806	0.806	0.806	0.806	0.598
Esm1B XGBoost	Anti gram (-)	0.882	0.882	0.882	0.882	0.764
Esm1B ExtraTrees	Anti gram (+)	0.881	0.881	0.883	0.881	0.764
ProTrans t5 xlu50 RandomForest	Anti inflamatory	0.886	0.886	0.891	0.886	0.777
ProTrans BERT ExtraTrees	Anti malarial	0.778	0.778	0.778	0.778	0.556
ProTrans t5 Uniref HistGradientBoosting	Anti-mammalian cell	0.899	0.899	0.899	0.899	0.798
Esm1B ExtraTrees	Anti methicillin-resistant S. aureus	0.875	0.876	0.884	0.875	0.753
ProTrans t5 xlu50 RandomForest	Anti oxidative	0.816	0.815	0.815	0.816	0.62
ProTrans T5-BDF XGBoost	Antibacterial	0.92	0.918	0.918	0.92	0.758

ProTrans t5 xlu50 ExtraTrees	Anti fungal	0.834	0.834	0.835	0.834	0.667
ProTrans t5 Uniref ExtraTrees	Antimicrobial	0.881	0.881	0.883	0.881	0.764
Esm1B ExtraTrees	Antiparasitic	0.844	0.844	0.848	0.844	0.692
ProTrans t5 Uniref RandomForest	Antiviral	0.785	0.785	0.786	0.785	0.57
ProTrans t5 xlu50 HistGradientBoosting	Anuran defense	0.926	0.926	0.926	0.926	0.852
ProTrans t5 Uniref ExtraTrees	Blood-brain barrier-penetrating	0.788	0.779	0.852	0.788	0.64
ProTrans t5 xlu50 HistGradientBoosting	Cell-cell communication	0.898	0.898	0.908	0.898	0.806
ProTrans ALBERT ExtraTrees	Cell-penetrating	0.863	0.863	0.863	0.863	0.724
Esm1B ExtraTrees	Drug delivery vehicle	0.88	0.88	0.88	0.88	0.76
ProTrans t5 xlu50 ExtraTrees	Neuropeptide	0.87	0.869	0.873	0.87	0.742
ProTrans ALBERT ExtraTrees	Quorum sensing	0.9	0.901	0.92	0.9	0.816

The area under the curve (AUC) was estimated using the independent dataset and employed as input for the classification of unknown peptide sequences. Table **S4** summarizes the AUC score for each trained model.

Supplementary Table S4: Summary Area under the curve (AUC) calculated using the independent dataset for each trained model

**Activity**	**AUC**
Anti angiogenic	0.8307
Anti diabetic	0.8848
Anti gram (-)	0.9230
Anti gram (+)	0.9227
Anti inflamatory	0.9197
Anti malarial	0.8833
Anti mammalian cell	0.9411
Anti Methicillin-resistant S. aureus	0.9107
Anti oxidative	0.8751
Antibacterial	0.7911
Antifungal	0.8904
Antimicrobial	0.9213
Antiparasitic	0.8908
Antiviral	0.6777
Anuran defense	0.9492
Blood brain barrier penetrating	0.8368
Cell cell communication	0.9439
Cell penetrating	0.9113
Drug delivery vehicle	0.9282
Neuropeptide	0.8988
Quorum sensing	0.9166

S7 Frequency of Residues for generated AMP sequences

Supplementary Table S5: Average frequency of amino acids in the studied sequences.

Residue	Peptide Atlas database	Raw AMP database	Trained VAE (generated)	AMP VAE (gen. pre-trained)	non-AMP VAE (gen. pre-trained)
A	0.073	0.069	0.084	0.069	0.071
C	0.018	0.046	0.055	0.156	0.143
D	0.031	0.025	0.021	0.016	0.017
E	0.044	0.033	0.062	0.055	0.06
F	0.05	0.046	0.033	0.021	0.02
G	0.077	0.077	0.074	0.15	0.151
H	0.022	0.021	0.012	0.003	0.002
I	0.074	0.06	0.035	0.013	0.013
N	0.035	0.03	0.029	0.019	0.018
K	0.085	0.115	0.108	0.138	0.14
L	0.116	0.106	0.139	0.116	0.128
M	0.023	0.014	0.025	0.002	0.002
P	0.04	0.045	0.033	0.039	0.036
Q	0.035	0.025	0.03	0.008	0.007
R	0.058	0.087	0.07	0.043	0.043
S	0.051	0.049	0.056	0.059	0.058
T	0.042	0.036	0.032	0.035	0.034
V	0.08	0.057	0.066	0.038	0.041
W	0.014	0.035	0.015	0.004	0.004
Y	0.03	0.024	0.023	0.014	0.013

S8 Deep learning architectures explored

Four deep learning architectures were explored to train predictive models for functional biological activities and to compare the performance of the proposed pipeline. These architectures include Convolutional Neural Networks (CNN), Bi-Long Short-Term Memory (LSTM), Bi-Gated Recurrent Unit (GRU), and a hybrid architecture combining CNN with LSTM. These architectures, inspired by previous work by the group (10), are summarized below. The binary cross-entropy loss function and the Adam optimizer were used across all architectures. The models were trained for 30 epochs with an early stopping criterion set at 10 epochs. The TensorFlow framework was utilized for the implementation and training of the classification models. The source code and full details of the architectures are available on the GitHub project `https://github.com/ProteinEngineering-PESB2/amp_class_ml`.

The training process utilized an embedding representation strategy for the proposed models for each task. The dataset was divided into training, validation, and independent test sets, with the same random seed used for hyperparameter tuning to ensure consistency.

Table **S6** summarizes the performance during the validation process for the 21 tasks. The results show that in tasks like Anti-Angiogenic and Neuropeptide classification, deep learning models perform worse than traditional methods. However, for tasks such as anti-diabetic, anti-oxidative, antifungal, and antiviral, deep learning approaches show superior performance. For the remaining tasks, there are no clear differences between traditional methods and deep learning models. In most cases, CNN-based architectures achieve the highest performance compared to LSTM or GRU architectures. The combination of CNN with LSTM does not significantly improve model precision. There is no clear preference for any specific architecture, presenting a challenge for future generalization of the implemented models.

Supplementary Table S6: Summary training process using deep learning architectures

Task	Current precision	CNN-1D	Bi-LSTM	Bi-GRU	CNN-1D/LSTM
Anti angiogenic	(0.74, 0.76)	0.71	0.74	0	0.74
Anti diabetic	(0.81, 0.82)	**0.88**	0.8	0.55	**0.85**
Anti gram (-)	(0.88, 0.89)	0.89	0.88	0.86	0.88
Anti gram (+)	(0.88, 0.88)	**0.9**	0.89	0.89	0.89

Anti-inflammatory	(0.87, 0.87)	**0.89**	0.84	0.84	**0.89**
Anti malarial	(0.83, 0.84)	**0.89**	**0.89**	0	0
Anti mammalian cell	(0.90, 0.90)	**0.91**	**0.91**	**0.91**	**0.92**
Anti methicillin-resistant S. aureus	(0.87, 0.87)	**0.91**	0.86	0	0.85
Anti oxidative	(0.75, 0.76)	**0.82**	0.75	0	0.75
Antibacterial	(0.89, 0.89)	**0.94**	**0.94**	0.77	**0.94**
Antifungal	(0.82, 0.82)	**0.86**	0.82	0.78	**0.83**
Antimicrobial	(0.87, 0.88)	**0.91**	**0.9**	**0.9**	**0.89**
Antiparasitic	(0.83, 0.83)	**0.86**	**0.85**	0.8	**0.85**
Antiviral	(0.80, 0.81)	**0.84**	**0.82**	0.77	**0.83**
Anuran defense	(0.90, 0.90)	**0.93**	**0.91**	**0.91**	**0.92**
Blood-brain barrier-penetrating	(0.78, 0.79)	**0.89**	0.63	0.52	**0.88**
Cell-cell communication	(0.90, 0.90)	**0.93**	**0.92**	**0.93**	**0.92**
Cell-penetrating	(0.87, 0.87)	**0.9**	0.87	0	0.85
Neuropeptide	(0.86, 0.86)	0.85	0.84	0.8	0.84
Quorum sensing	(0.87, 0.88)	**1**	0.79	0.6	0
Drug delivery vehicle	(0.84, 0.85)	0.85	**0.86**	**0.87**	0.84

Table **S7** summarizes the performance of the deep learning architectures for the 21 evaluated tasks during the testing stage. In most cases, the precision of the deep learning models is lower than that of the models implemented using the proposed pipeline. The differences observed between validation and testing performances suggest overfitting during the training process. However, for tasks such as antimicrobial, cell-cell communication, cell-penetrating, antiviral, and anti-mammalian cell detection, CNN-based deep learning models achieve higher performance than the implemented models in this work. These tasks also include datasets with the highest number of examples, except for the antibacterial dataset, indicating that deep learning approaches generalize well when the number of examples is substantial.

To address generalization problems in low-N datasets, exploring transfer learning and fine-tuning approaches could be beneficial. These strategies could help train generalizable predictive models for tasks like quorum sensing and anuran defense. Future work should focus on investigating these deep learning-based strategies to enhance model performance and generalization.

Supplementary Table S7: Summary precision performances for explored deep learning architectures during testing process

Task	Current precision	CNN-1D	Bi-LSTM	Bi-GRU	CNN-1D/LSTM
Anti angiogenic	**0.82**	0.63	0.65	0	0.65
Anti diabetic	**0.81**	0.72	0.71	0.5	0.75
Anti gram (-)	0.88	**0.9**	**0.89**	0.88	**0.89**
Anti gram (+)	0.88	**0.89**	0.88	0.87	0.88
Anti-inflammatory	**0.89**	0.88	0.85	0.82	0.85
Anti malarial	0.78	0.73	**0.89**	0	0
Anti mammalian cell	0.9	**0.91**	**0.91**	**0.92**	**0.92**
Anti methicillin-resistant S. aureus	**0.9**	0.83	0.83	0	0.78
Anti oxidative	**0.82**	0.7	0.69	0	0.63
Antibacterial	**0.92**	0.82	0.81	0.5	0.82
Antifungal	**0.84**	**0.84**	0.81	0.74	0.83
Antimicrobial	0.88	**0.9**	**0.9**	**0.9**	0.8
Antiparasitic	**0.85**	0.84	**0.85**	0.76	0.83
Antiviral	0.79	**0.82**	0.78	0.77	**0.81**
Anuran defense	**0.93**	0.84	0.86	0.85	0.85
Blood-brain barrier-penetrating	**0.85**	0.84	0.72	0.5	0.81
Cell-cell communication	0.91	**0.92**	**0.92**	**0.92**	**0.94**
Cell-penetrating	0.86	**0.91**	0.84	0	0.83
Neuropeptide	**0.87**	0.86	0.85	0.84	0.84
Quorum sensing	**0.87**	0.8	0.8	0.5	0

Drug delivery vehicle	**0.92**	0.83	0.87	0.85	0.87

S9 Working with SMOTE as oversampling strategy

Working with unbalanced datasets necessitates the use of oversampling or undersampling strategies to achieve balance. In this work, we applied undersampling strategies because techniques like SMOTE do not ensure that the new examples are valid peptide sequences or that they accurately represent the class we need to balance. This presents a significant challenge in developing classification models for peptide sequences. Nevertheless, we explored SMOTE as an oversampling approach for the anuran defence and quorum sensing peptide detection datasets.

For anuran defense detection, the results from the exploration stage showed similar performance to the confidence interval obtained with undersampling strategies. However, for quorum sensing models, the performance decreased compared to the results obtained with undersampling approaches.

Methods like Variational Autoencoders (VAE) or similar techniques, which learn about the distribution of the latent space, could be promising alternatives for addressing unbalanced datasets. We plan to explore these approaches in our future work on building predictive models for low-N datasets.

S10 Property distribution analysis of AMP discovered peptide sequences and generated *de novo* AMP peptide sequences

Nine properties were evaluated to compare the annotated peptide sequences extracted from Peptipedia v2.0 (4). These properties were estimated using the physicochemical characterization module available on Peptipedia v2.0. Figures **S1**, **S2**, **S3**, **S4**, **S5**, **S6**, **S7**, **S8**, and **S9** illustrate these properties. Notable differences are observed in properties such as molecular weight, charge, and instability.

A Kruskal-Wallis statistical test combined with a post-hoc analysis was applied to detect differences between the various sources and strategies evaluated in this work. With the exception of molecular weight, all properties were similar across all sources. In contrast, Peptide Atlas showed a different distribution for molecular weight compared to raw data and generated AMP peptide sequences through VAE approaches.

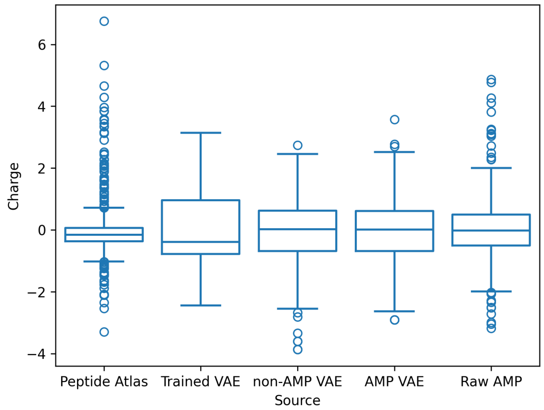

Supplementary Figure S1: Charge distribution for the explored and de novo generated peptide sequences analysed in this work.

References

[1] Agrawal, P., Bhalla, S., Chaudhary, K., Kumar, R., Sharma, M., and Raghava, G. P. (2018). In silico approach for prediction of antifungal peptides. *Frontiers in microbiology*, 9:318353.

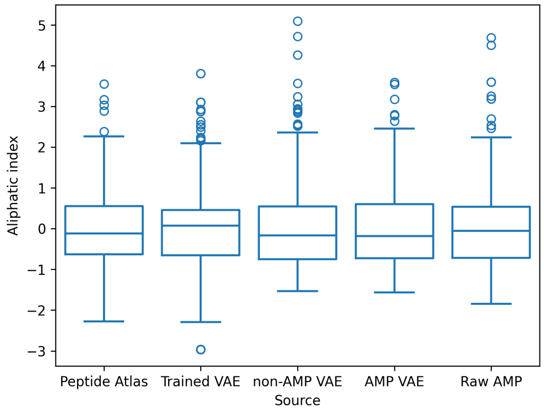

Supplementary Figure S2: Aliphatic index distribution for the explored and de novo generated peptide sequences analysed in this work.

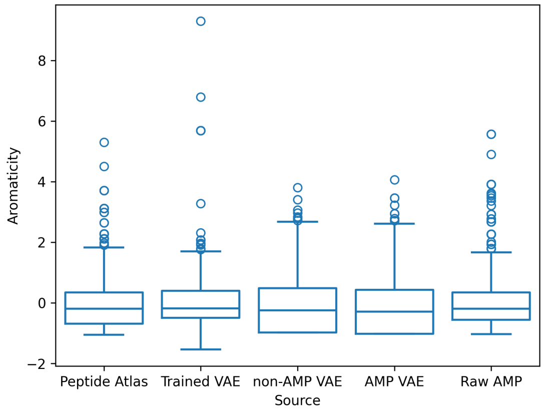

Supplementary Figure S3: Aromaticity index distribution for the explored and de novo generated peptide sequences analysed in this work.

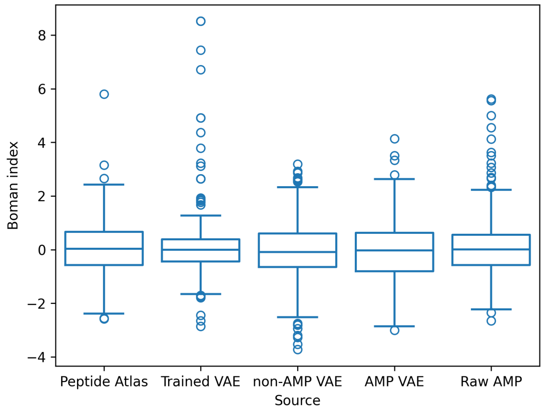

Supplementary Figure S4: Boman index distribution for the explored and de novo generated peptide sequences analysed in this work.

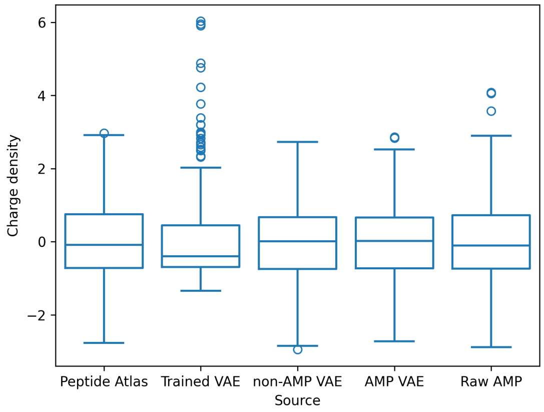

Supplementary Figure S5: Charge density distribution for the explored and de novo generated peptide sequences analysed in this work.

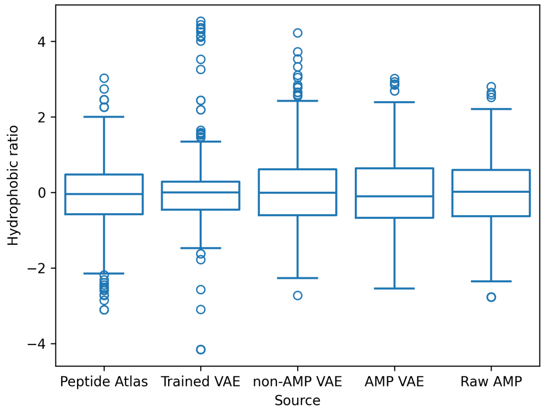

Supplementary Figure S6: Hydrophobicity ratio distribution for the explored and de novo generated peptide sequences analysed in this work.

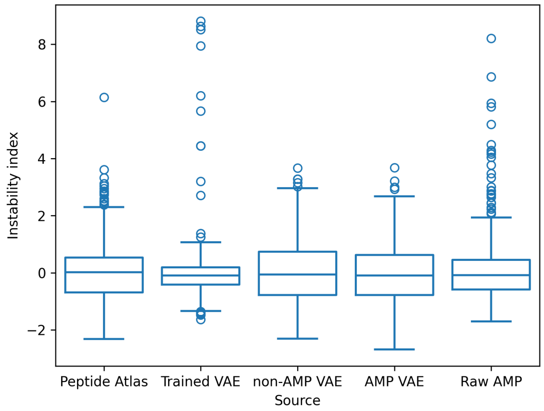

Supplementary Figure S7: Instability index distribution for the explored and de novo generated peptide sequences analysed in this work.

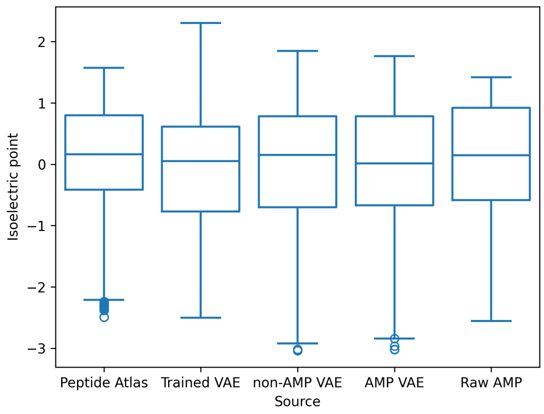

Supplementary Figure S8: Isoelectric point distribution for the explored and de novo generated peptide sequences analysed in this work.

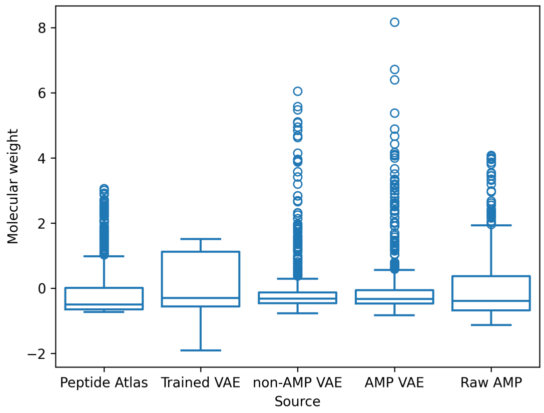

Supplementary Figure S9: Molecular weight distribution for the explored and de novo generated peptide sequences analysed in this work.

[2] Bajiya, N., Choudhury, S., Dhall, A., and Raghava, G. P. (2024). Antibp3: A method for predicting antibacterial peptides against gram-positive/negative/variable bacteria. *Antibiotics*, 13(2):168.

[3] Burdukiewicz, M., Sidorczuk, K., Rafacz, D., Pietluch, F., Chilimoniuk, J., Rødiger, S., and Gagat, P. (2020). Proteomic screening for prediction and design of antimicrobial peptides with ampgram. *International journal of molecular sciences*, 21(12):4310.

[4] Cabas, G., Daza, A., Soto, N., Garrido, V., Alvarez, D., Navarrete, M., Sarmiento-Varon, L., Sepulveda, J., Davari, M. D., Cadet, F., Á., O.-N., R., U.-P., and D., M.-O. (2024). Peptipedia v2. 0: A peptide sequence database and user-friendly web platform. a major update. *bioRxiv*, pages 2024–07.

[5] Chowdhury, A. S., Reehl, S. M., Kehn-Hall, K., Bishop, B., and Webb-Robertson, B.-J. M. (2020). Better understanding and prediction of antiviral peptides through primary and secondary structure feature importance. *Scientific reports*, 10(1):19260.

[6] Chung, C.-R., Kuo, T.-R., Wu, L.-C., Lee, T.-Y., and Horng, J.-T. (2020). Characterization and identification of antimicrobial peptides with different functional activities. *Briefings in bioinformatics*, 21(3):1098–1114.

[7] Dong, G., Zheng, L., Huang, S., Gao, J., and Zuo, Y. (2021). Amino acid reduction can help to improve the identification of antimicrobial peptides and their functional activities. front genet 12.

[8] Du, Z., Ding, X., Xu, Y., and Li, Y. (2023). Unidl4biopep: a universal deep learning architecture for binary classification in peptide bioactivity. *Briefings in Bioinformatics*, 24(3):bbad135.

[9] Feng, J., Sun, M., Liu, C., Zhang, W., Xu, C., Wang, J., Wang, G., and Wan, S. (2024). Samp: Identifying antimicrobial peptides by an ensemble learning model based on proportionalized split amino acid composition. *bioRxiv*, pages 2024–04.

[10] Fernández, D., Olivera-Nappa, Á., Uribe-Paredes, R., and Medina-Ortiz, D. (2023). Exploring machine learning algorithms and protein language models strategies to develop enzyme classification systems. In *International Work-Conference on Bioinformatics and Biomedical Engineering*, pages 307–319. Springer.

[11] Grønning, A. G., Kacprowski, T., and Scheele, C. (2021). Multipep: a hierarchical deep learning approach for multi-label classification of peptide bioactivities. *Biology Methods and Protocols*, 6(1):bpab021.

[12] Guan, J., Yao, L., Xie, P., Chung, C.-R., Huang, Y., Chiang, Y.-C., and Lee, T.-Y. (2024). A two-stage computational framework for identifying antiviral peptides and their functional types based on contrastive learning and multi-feature fusion strategy. *Briefings in Bioinformatics*, 25(3):bbae208.

[13] Gull, S., Shamim, N., and Minhas, F. (2019). Amap: Hierarchical multi-label prediction of biologically active and antimicrobial peptides. *Computers in biology and medicine*, 107:172–181.

[14] Joseph, S., Karnik, S., Nilawe, P., Jayaraman, V. K., and Idicula-Thomas, S. (2012). Classamp: a prediction tool for classification of antimicrobial peptides. *IEEE/ACM Transactions on Computational Biology and Bioinformatics*, 9(5):1535–1538.

[15] Kavousi, K., Bagheri, M., Behrouzi, S., Vafadar, S., Atanaki, F. F., Lotfabadi, B. T., Ariaeenejad, S., Shockravi, A., and Moosavi-Movahedi, A. A. (2020). Iampe: Nmr-assisted computational prediction of antimicrobial peptides. *Journal of Chemical Information and Modeling*, 60(10):4691–4701.

[16] Lata, S., Mishra, N. K., and Raghava, G. P. (2010). Antibp2: improved version of antibacterial peptide prediction. *BMC bioinformatics*, 11:1–7.

[17] Lee, H., Lee, S., Lee, I., and Nam, H. (2023). Amp-bert: Prediction of antimicrobial peptide function based on a bert model. *Protein Science*, 32(1):e4529.

[18] Li, C., Warren, R. L., and Birol, I. (2023). Models and data of amplify: a deep learning tool for antimicrobial peptide prediction. *BMC Research Notes*, 16(1):11.

[19] Li, J., Pu, Y., Tang, J., Zou, Q., and Guo, F. (2020). Deepavp: a dual-channel deep neural network for identifying variable-length antiviral peptides. *IEEE journal of biomedical and health informatics*, 24(10):3012–3019.

[20] Lin, T.-T., Sun, Y.-Y., Wang, C.-T., Cheng, W.-C., Lu, I.-H., Lin, C.-Y., and Chen, S.-H. (2022). Ai4avp: an antiviral peptides predictor in deep learning approach with generative adversarial network data augmentation. *Bioinformatics Advances*, 2(1):vbac080.

[21] Lin, W. and Xu, D. (2016). Imbalanced multi-label learning for identifying antimicrobial peptides and their functional types. *Bioinformatics*, 32(24):3745–3752.

[22] Lv, H., Yan, K., and Liu, B. (2023). Tppred-le: therapeutic peptide function prediction based on label embedding. *BMC biology*, 21(1):238.

[23] Medina-Ortiz, D., Cabas-Mora, G., Moya-Barria, I., Soto-Garcia, N., and Uribe-Paredes, R. (2024). Rudeus, a machine learning classification system to study dna-binding proteins. *bioRxiv*, pages 2024–02.

[24] Meher, P. K., Sahu, T. K., Saini, V., and Rao, A. R. (2017). Predicting antimicrobial peptides with improved accuracy by incorporating the compositional, physico-chemical and structural features into chou's general pseaac. *Scientific reports*, 7(1):42362.

[25] Pinacho-Castellanos, S. A., García-Jacas, C. R., Gilson, M. K., and Brizuela, C. A. (2021). Alignment-free antimicrobial peptide predictors: improving performance by a thorough analysis of the largest available data set. *Journal of Chemical Information and Modeling*, 61(6):3141–3157.

[26] Pirtskhalava, M., Amstrong, A. A., Grigolava, M., Chubinidze, M., Alimbarashvili, E., Vishnepolsky, B., Gabrielian, A., Rosenthal, A., Hurt, D. E., and Tartakovsky, M. (2021). Dbaasp v3: database of antimicrobial/cytotoxic activity and structure of peptides as a resource for development of new therapeutics. *Nucleic acids research*, 49(D1):D288–D297.

[27] Randou, E. G., Veltri, D., and Shehu, A. (2013). Binary response models for recognition of antimicrobial peptides. In *Proceedings of the International Conference on Bioinformatics, Computational Biology and Biomedical Informatics*, pages 76–85.

[28] Sharma, R., Shrivastava, S., Kumar Singh, S., Kumar, A., Saxena, S., and Kumar Singh, R. (2021). Deep-abppred: identifying antibacterial peptides in protein sequences using bidirectional lstm with word2vec. *Briefings in Bioinformatics*, 22(5):bbab065.

[29] Sharma, R., Shrivastava, S., Kumar Singh, S., Kumar, A., Saxena, S., and Kumar Singh, R. (2022). Deep-afppred: identifying novel antifungal peptides using pretrained embeddings from seq2vec with 1dcnn-bilstm. *Briefings in Bioinformatics*, 23(1):bbab422.

[30] Simeon, S., Li, H., Win, T. S., Malik, A. A., Kandhro, A. H., Piacham, T., Shoombuatong, W., Nuchnoi, P., Wikberg, J. E., Gleeson, M. P., et al. (2017). Pepbio: Predicting the bioactivity of host defense peptides. *RSC advances*, 7(56):35119–35134.

[31] Singh, V., Shrivastava, S., Kumar Singh, S., Kumar, A., and Saxena, S. (2022). Stable-abppred: a stacked ensemble predictor based on bilstm and attention mechanism for accelerated discovery of antibacterial peptides. *Briefings in Bioinformatics*, 23(1):bbab439.

[32] Thakur, N., Qureshi, A., and Kumar, M. (2012). Avppred: collection and prediction of highly effective antiviral peptides. *Nucleic acids research*, 40(W1):W199–W204.

[33] Torrent, M., Di Tommaso, P., Pulido, D., Nogués, M. V., Notredame, C., Boix, E., and Andreu, D. (2012). Ampa: an automated web server for prediction of protein antimicrobial regions. *Bioinformatics*, 28(1):130–131.

[34] Veltri, D., Kamath, U., and Shehu, A. (2018). Deep learning improves antimicrobial peptide recognition. *Bioinformatics*, 34(16):2740–2747.

[35] Wang, P., Hu, L., Liu, G., Jiang, N., Chen, X., Xu, J., Zheng, W., Li, L., Tan, M., Chen, Z., et al. (2011). Prediction of antimicrobial peptides based on sequence alignment and feature selection methods. *PloS one*, 6(4):e18476.

[36] Wang, R., Wang, T., Zhuo, L., Wei, J., Fu, X., Zou, Q., and Yao, X. (2024). Diff-amp: tailored designed antimicrobial peptide framework with all-in-one generation, identification, prediction and optimization. *Briefings in Bioinformatics*, 25(2):bbae078.

[37] Wang, S.-C. (2024). E-cleap: An ensemble learning model for efficient and accurate identification of antimicrobial peptides. *Plos one*, 19(5):e0300125.

[38] Xiao, X., Shao, Y.-T., Cheng, X., and Stamatovic, B. (2021). iamp-ca2l: a new cnn-bilstm-svm classifier based on cellular automata image for identifying antimicrobial peptides and their functional types. *Briefings in bioinformatics*, 22(6):bbab209.

[39] Xiao, X., Wang, P., Lin, W.-Z., Jia, J.-H., and Chou, K.-C. (2013). iamp-2l: a two-level multi-label classifier for identifying antimicrobial peptides and their functional types. *Analytical biochemistry*, 436(2):168–177.

[40] Xing, W., Zhang, J., Li, C., Huo, Y., and Dong, G. (2024). iamp-attenpred: a novel antimicrobial peptide predictor based on bert feature extraction method and cnn-bilstm-attention combination model. *Briefings in Bioinformatics*, 25(1):bbad443.

[41] Yan, J., Bhadra, P., Li, A., Sethiya, P., Qin, L., Tai, H. K., Wong, K. H., and Siu, S. W. (2020). Deep-ampep30: improve short antimicrobial peptides prediction with deep learning. *Molecular Therapy-Nucleic Acids*, 20:882–894.

[42] Yan, W., Tang, W., Wang, L., Bin, Y., and Xia, J. (2022). Prmftp: Multi-functional therapeutic peptides prediction based on multi-head self-attention mechanism and class weight optimization. *PLoS computational biology*, 18(9):e1010511.

[43] Yao, L., Guan, J., Xie, P., Chung, C.-R., Deng, J., Huang, Y., Chiang, Y.-C., and Lee, T.-Y. (2024). Ampactipred: A three-stage framework for predicting antibacterial peptides and activity levels with deep forest. *Protein Science*, 33(6):e5006.

[44] Yao, L., Zhang, Y., Li, W., Chung, C.-R., Guan, J., Zhang, W., Chiang, Y.-C., and Lee, T.-Y. (2023). D eepafp: An effective computational framework for identifying antifungal peptides based on deep learning. *Protein Science*, 32(10):e4758.

[45] Youmans, M., Spainhour, J. C. G., and Qiu, P. (2020). Classification of antibacterial peptides using long short-term memory recurrent neural networks. *IEEE/ACM Transactions on Computational Biology and Bioinformatics*, 17(4):1134–1140.

[46] Zhang, J., Yang, L., Tian, Z., Zhao, W., Sun, C., Zhu, L., Huang, M., Guo, G., and Liang, G. (2021). Large-scale screening of antifungal peptides based on quantitative structure–activity relationship. *ACS Medicinal Chemistry Letters*, 13(1):99–104.

[Zhang et al.] Zhang, W., Xia, E., Dai, R., Tang, W., Bin, Y., and Xia, J. Predapp: predicting anti-parasitic peptides with undersampling and ensemble approaches. interdiscip sci comput life sci 2022; 14: 258–68.
